# Occurrence and Antimicrobial Resistance of *Acinetobacter* spp. in Processing Environments of Slaughterhouses and Meat Processing Facilities

**DOI:** 10.3390/foods15071243

**Published:** 2026-04-05

**Authors:** Alba Puente, Rebeca Cordero-García, Elena Fernández-Trapote, Victoria Crespo-Torbado, Márcia Oliveira, Mercedes López, Miguel Prieto, Avelino Alvarez-Ordóñez, José F. Cobo-Díaz

**Affiliations:** 1 Department of Food Science and Technology, *Universidad de León*, 24071 *León*, Spain; apueb@unileon.es (A.P.); rcorg@unileon.es (R.C.-G.); efert@unileon.es (E.F.-T.); vcret@unileon.es (V.C.-T.); msouo@unileon.es (M.O.); mmlopf@unileon.es (M.L.); miguel.prieto@unileon.es (M.P.); aalvo@unileon.es (A.A.-O.); 2 Institute of Food Science and Technology (ICTAL), *Universidad de León*, 24071 *León*, Spain

**Keywords:** *Acinetobacter* spp., meat processing environments, carcasses, surfaces, antimicrobial resistance, MDR

## Abstract

Several species of the genus *Acinetobacter* are nosocomial pathogens with a well-documented ability to acquire resistance to multiple antibiotics. Although *Acinetobacter* is one of the most abundant genera in meat processing environments, data on this genus outside of clinical environments remains limited. The objective of this study was to ascertain the prevalence, diversity and antimicrobial resistance profile of *Acinetobacter* spp. in 200 samples collected from food contact surfaces, non-food contact surfaces, carcasses and final meat cuts across five pork, chicken and beef processing facilities, each comprising physically connected slaughterhouses and meat processing plants. *Acinetobacter* spp. were detected in 80% (95% CI = 71–87%) and 70% (95% CI = 60–79%) of samples from slaughterhouses and processing plants, respectively. The facilities harboured a wide diversity of *Acinetobacter* species, with 27 different species identified. *Acinetobacter baumannii* was the species most frequently detected. Whole-genome sequencing of 18 *Acinetobacter* spp. isolates revealed the presence of ARGs conferring resistance to beta-lactams, tetracyclines and aminoglycosides, and disclosed phylogenetic relationships with isolates from fresh meat. Phenotypic resistance to beta-lactams, fluoroquinolones, aminoglycosides, folate pathway inhibitors and/or tetracyclines was observed in 77.8% of the sequenced isolates, with 44.4% classified as multidrug-resistant. These findings identify meat processing environments as an important reservoir of *Acinetobacter* spp. and highlight the need for further investigation to prevent the dissemination of antimicrobial-resistant strains.

## 1. Introduction

Antimicrobial resistance (AMR) is considered among the top 10 global public health and socioeconomic problems [1]. There is evidence that food processing environments constitute an important source for AMR, and certain processes may promote the emergence and transmission of antimicrobial resistance genes (ARGs) [2,3]. The extensive and inappropriate use of antimicrobials in livestock has induced a selective pressure leading to the spread of antimicrobial-resistant bacteria in meat processing facilities [4,5], which may persist on food contact surfaces (FCSs) and non-food contact surfaces (NFCSs) in these types of facilities [6,7,8]. These surfaces can then serve as a source of contamination with antimicrobial-resistant bacteria for carcasses and meat during the processes conducted in slaughterhouses and meat processing plants. Remarkably, the occurrence of antimicrobial-resistant bacteria in meat may constitute a possible risk to consumers through the transfer of ARGs to commensal and/or pathogenic human gut bacteria through horizontal gene transfer [9].

Previous metagenomic investigations have identified *Acinetobacter*, *Pseudomonas* and *Staphylococcus*, among other genera, as the most prevalent bacterial genera associated with AMR in slaughterhouses, meat processing environments and meat products [7,10,11]. Moreover, a high prevalence of *Acinetobacter* spp. isolates has been previously observed in fresh meat [12,13]. Some *Acinetobacter* species are associated with nosocomial infections and resistance to multiple antibiotics [14]. Among them, *Acinetobacter baumannii* is the species most frequently related to multidrug-resistant (MDR) infections [15]. Notably, carbapenem-resistant *A. baumannii* isolates have been categorised as critical-priority pathogens in the search for new effective antibiotics [16] and are included in the ESKAPE (*Enterococcus faecium*, *Staphylococcus aureus*, *Klebsiella pneumoniae*, *Acinetobacter baumannii*, *Pseudomonas aeruginosa*, and *Enterobacter* spp.) group of highly virulent and MDR nosocomial pathogens [17]. Additionally, a considerable number of other *Acinetobacter* species have also been involved in human infections, such as *A. nosocomialis* and *A. pittii*, members of the *Acinetobacter calcoaceticus-Acinetobacter baumannii* (ACB) complex, which encompasses phenotypically related species [14,17,18].

Considering this evidence, the aim of the current study was to comprehensively investigate the possible role of meat processing environments as a reservoir of antimicrobial-resistant *Acinetobacter* spp. In order to achieve this, the following specific objectives were identified: (i) to determine the occurrence of *Acinetobacter* spp. on FCSs and NFCSs throughout processing lines, as well as on carcasses and final meat cuts in slaughterhouses and meat processing plants; (ii) to characterise the diversity of *Acinetobacter* species in such environments; (iii) to phenotypically and genotypically characterise various *Acinetobacter* spp. isolates obtained from these samples, including the identification of ARGs and their genomic location, as well as antimicrobial susceptibility testing of selected strains; and (iv) to elucidate the phylogenetic relationships between *Acinetobacter* spp. isolates obtained in the current study and those previously recovered from fresh meat samples in local markets by our research group [13].

## 2. Materials and Methods

### 2.1. Sampling Plan

Five meat processing industries, with slaughterhouse and processing plant physically connected, were sampled between March 2022 and May 2023. These included pork (*n* = 2; labelled in figures as pork_1 and pork_2), chicken (*n* = 2; chicken_1 and chicken_2), and beef (*n* = 1; beef) processing facilities located in Spain. The slaughterhouses and connected meat processing plants were sampled during routine operations at the beginning of the workday on a single visit. For each slaughterhouse, a total of 20 samples were collected. This included 8 samples from FCSs (except in pork_1 where it was only possible to take 7 different samples), 8 samples from NFCSs (except in pork_1 where 9 samples were collected) and 4 samples from raw materials (2 carcasses collected at the end of the dirty zone, and 2 carcasses at the end of the slaughter line (clean zone) before entering the cooling room). FCS samples encompassed knives, hooks, trays, and water from the scalding tanks and from the de-hairing equipment (Table 1). NFCSs included drains, floors and walls (Table 1). FCS and NFCS samples were taken from the dirty and clean zones. In each processing plant, 20 samples were also collected, including 10 samples from FCSs, 6 from NFCSs, and 4 from raw materials (2 carcasses after cooling and at the start of the processing process, and 2 final meat cuts such as breasts, sirloin, etc.). NFCSs encompassed drains, floors and walls (Table 2). Thus, a total of 200 samples (100 from slaughterhouses and 100 from their corresponding processing plants) were collected. Samples were taken using Whirl–Pak Hydrated PolyProbe swabs (Whirl–Pak, Fort Atkinson, WI, USA). When adequate surface area was available (e.g., floors, walls, and carcasses), approximately 1 m^2^ was sampled. For smaller or irregular items (e.g., knives, drains, and final product), individual units were swabbed separately (one knife, one drain, etc.). During sampling, disposable protective clothing, including footwear, coats, caps, facial masks and gloves (replaced between samples), was used. Sampling bags were stored in ice-cooled containers and transported to the laboratory for processing within 12 h of collection.

### 2.2. Isolation of Acinetobacter spp.

Under aseptic conditions, all swabs were suspended in 15 mL of Buffer Peptone Water (0.1%) (BPW, Merck KGaA, Darmstadt, Germany), supplemented with 1 mg/L vancomycin, 1.5 mg/L cefsulodin and 5 mg/L cephradine (Sigma-Aldrich, Saint Louis, MO, USA) to reduce the growth of Gram-positive bacteria, and then homogenised using a Stomacher (IUL Instruments, Barcelona, Spain) at maximum speed for 2 min. The isolation of *Acinetobacter* spp. was carried out according to Puente et al. [13] by using the selective medium CHROMagar™ Acinetobacter medium (CHROMagar, Paris, France), and the same medium supplemented with CHROMagar™ MDR Selective Supplement (CHROMagar) to isolate carbapenem-resistant *Acinetobacter* spp. In order to isolate mesophilic and psychrotolerant species of *Acinetobacter* spp., two different enrichment and incubation methods were considered. Psychrotolerant species were isolated by spreading 100 µL aliquots of the homogenate BPW onto both unsupplemented and supplemented agar media (two replicates per medium) and incubating the plates for 10 days at 2 °C. To isolate mesophilic species, a non-selective pre-enrichment in BPW was performed by incubating the sampling bags containing the swabs at 35 °C for 24 h. After incubation, 10 µL of the solution was streaked onto both unsupplemented and supplemented agar plates (two replicates per medium), followed by incubation at 35 °C for 24 h.

### 2.3. Identification of Acinetobacter spp.

In both cases, the identity of presumptive *Acinetobacter* spp. colonies was confirmed by Matrix-Assisted Laser Desorption–Ionisation Time-of-Flight Mass Spectrometry (MALDI-TOF MS, Microflex LT model, Bruker-Daltonics, Billerica, MA, USA) according to Bruker specifications. Briefly, one red colony by morphology per sample and growth medium were selected and streaked onto Brain Heart Infusion agar plates (BHI, Merck KGaA), which were incubated for two days at 20 °C for psychrotolerant isolates or 24 h at 35 °C for mesophilic isolates. Afterwards, a single colony was smeared using a sterile pipette tip onto a well of the MSP96 Bruker steel plate. Then, 1 μL of matrix solution (α-cyano-4-hydroxycinnamic acid, tri-fluoroacetic-acid (TFA) and acetonitrile) was added to the well, and the plate was left to dry for 5 min at room temperature in a laminar flow cabinet. For calibration, the Bruker BTS standard (*Escherichia coli* DH5 α extract) was added to one well of the plate. For bacterial identification, the MALDI Biotyper software (version 4.1.80), with the spectra reference library (v.13.0.0.2) (Bruker Daltonics), was employed. Isolates identified as members of the *Acinetobacter* genus with a log score value > 1.70 (reliable identification at genus level) were conserved at −80 °C in Cryoinstant Mixed vials (VWR, Radnor, PA, USA) for future analyses. In addition, *16S rRNA* gene analysis by Sanger sequencing was employed to confirm the identity of psychrotolerant isolates collected at refrigeration temperature that were previously identified by MALDI-TOF as members of the genus *Acinetobacter* but not classified at the species level. To amplify the *16S rRNA* gene, the primers 27F (AGA GTT TGA TCM TGG CTC AG) and 1492R (GGT TAC CTT GTT ACG ACTT) were used. PCR tests were performed using a ProFlex PCR System thermocycler (Applied Biosystems, Foster City, CA, USA) with the following conditions: 35-cycle amplification protocol consisting of 95 °C for 30 s, hybridisation at 57 °C for 30 s, and extension for 1 min at 72 °C. Finally, a 10 min extension at 72 °C was performed. The PCR products obtained were purified using the NucleoSpin Gel and PCR Clean-up kit (Macherey-Nagel, Düren, Germany) and sequenced using the BigDye Terminator (v.3.1) Cycle Sequencing Kit (Applied Biosystems) and an ABI 3500 automatic capillary sequencer (Applied Biosystems) following the manufacturer’s instructions. The sequences were analysed using the BioEdit biological sequence alignment editor programme.

### 2.4. Whole Genome Sequencing (WGS)

A total of 18 *Acinetobacter* spp. isolates were selected for WGS, comprising 10 mesophilic and eight psychrotolerant isolates (since the number of mesophilic isolates obtained was higher than the psychrotolerant isolates). The mesophilic group included eight *A. baumannii* and two *A. seifertii* isolates. *A. baumannii* was chosen due to its clinical relevance and high prevalence in our survey, while *A. seifertii*, the second most frequently found species, was included as a member of the ACB complex. Due to the prevalence among the psychrotolerant species being similar, we selected isolates from two of the most frequently detected species, including five *A. albensis* and two *A. johnsonii*, and one *A. terrestris*, which was only detected in one sample.

The DNA extraction was performed using the DNeasy Blood & Tissue Kit (Qiagen, Hilden, Germany) according to the manufacturer’s protocol for Gram-negative bacteria with a final elution with 100 µL of Buffer AE (10 mM Tris-Cl, 0.5 mM EDTA). The Qubit^®^ 3.0 Fluorometer and the dsDNA HS assay kit (Thermo Fisher Scientific, Waltham, MA, USA) were used for the quantification of DNA concentration.

The preparation of 150 bp paired-end sequencing libraries was undertaken using the Nextera XT DNA Library Preparation kit (Illumina Inc., San Diego, CA, USA), according to the manufacturer’s protocol. Sequencing was carried out using the NovaSeq 6000 Sequencing System (Macrogen Inc., Seoul, Korea). Trim Galore (v.0.6.6) (https://github.com/FelixKrueger/TrimGalore, accessed on 20 October 2025) was employed to remove adapters, low-quality reads (Phred score < 20), short reads (<75 bp), and reads with more than two ambiguous nucleotides, whereas Bowtie2 (v.2.3.4.3) [19] was used to remove contaminant DNA by deleting reads from the phiX174 Illumina spike-in (NCBI accession number NC_001422) and potential human contamination (using the GRCh38.p13 human genome, NCBI accession number GCF_000001405.39). The assembly of reads was conducted by SPAdes (v.3.15.2) [20], with the parameter *-k 55*, *75*, *97*. The quality of the obtained genomes was assessed using CheckM2 [21], and the taxonomic identification of genomes was confirmed using GTDB-Tk (v.1.7.0) [22]. The presence of ARGs was detected with BLASTn [23] against the ResFinder database (downloaded on 24 October 2022) [24] with a cut-off of 80% for both identity and gene coverage, while plasmids were identified using Platon [25]. To determine fluoroquinolone-resistance mechanisms, mutations in the *gyrA* sequence were analysed using a BLASTx [23] alignment of the respective *Acinetobacter* spp. genomes versus aminoacidic sequences from the reference genome *A. baumannii* strain K09–14 (GenBank: CP043953.1).

### 2.5. Multi-Locus Sequence Typing (MLST) and Phylogenetic Analysis of the Strains

MLST was performed using the mlst software (https://github.com/tseemann/mlst, accessed on 20 October 2025) on eight *A. baumannii* genomes isolated in the present study and 33 *A. baumannii* isolates previously obtained from retail fresh meat by our research group [13]. The *abaumannii* (Oxford scheme) and *abaumannii_2* (Pasteur scheme) typing schemes available from the PubMLST database (https://pubmlst.org/, accessed on 20 October 2025) were applied [26]. For the representation of results, a dendrogram based on Average Nucleotide Identity (ANI) values, obtained by dRep (v.2.6.6) [27], was constructed with the 18 genomes. Additionally, to determine the phylogenetic relationship between environmental and food *Acinetobacter* spp. isolates, three core-genome phylogenetic trees were constructed for *A. baumannii*, *A. seifertii* and *A. johnsonii* species. Each phylogenetic tree included eight *A. baumannii*, two *A. seifertii*, and two *A. johnsonii* isolates sequenced in the present study, together with 33 *A. baumannii*, two *A. seifertii*, and five *A. johnsonii* isolates previously recovered from fresh meat from supermarkets during an earlier study conducted by our research group [13]. To this end, the genomic sequences were annotated using Prokka (v.1.14.6) [28], and the core genes were compared using Panaroo (v.1.5.2) [29]. Finally, the phylogenetic trees were generated using FastTree (v.2.2.0) [30] by the neighbor-joining (NJ) method and the Jukes–Cantor model.

### 2.6. Antimicrobial Susceptibility Testing

The susceptibility of the 18 selected isolates to various antibiotics was tested by the broth microdilution method and Sensititre™ Gram Negative Non-Fermenters (NF) plates (Thermo Fisher Scientific), according to the manufacturer’s instructions. These plates included the following antibiotics and concentration ranges: amikacin (4–32 μg/mL), ampicillin-sulbactam (2/1–16/8 μg/mL), aztreonam (2–16 μg/mL), carbenicillin (32–256 μg/mL), cefepime (2–16 μg/mL), cefoperazone (4–32 μg/mL), cefotaxime (4–32 μg/mL), ceftazidime (1–16 μg/mL), ceftriaxone (4–32 μg/mL), chloramphenicol (2–16 μg/mL), ciprofloxacin (0.25–2 μg/mL), gentamicin (1–8 μg/mL), imipenem (1–8 μg/mL), levofloxacin (0.5–4 μg/mL), lomefloxacin (0.5–4 μg/mL), piperacillin (8–64 μg/mL), piperacillin-tazobactam (8/4–64/4 μg/mL), sulfisoxazole (256 μg/mL), tetracycline (1–8 μg/mL), ticarcillin (8–64 μg/mL), ticarcillin-clavulanic acid (16/2–128/2 μg/mL), tobramycin (1–8 μg/mL) and trimethoprim-sulfamethoxazole (0.5/9.5–4/76 μg/mL). After incubation for 24 h at 35 °C for mesophilic or 20 °C for psychrotolerant isolates, the minimum inhibitory concentration (MIC) of each antibiotic was determined visually based on the presence or absence of a bacterial pellet in each well. The lowest concentration of antibiotic without bacterial growth in the well was designated as the MIC. Moreover, in order to determine the susceptibility to the antibiotics mentioned, the European Committee on Antimicrobial Susceptibility Testing clinical breakpoints [31] were utilised, with the exception of cefotaxime, ceftazidime, piperacillin, tetracycline, piperacillin-tazobactam, ticarcillin-clavulanic acid, ampicillin-sulbactam, ceftriaxone, and cefepime, for which the Clinical and Laboratory Standards Institute [32] breakpoints were employed. Those isolates showing resistance to at least one antimicrobial in three or more antimicrobial categories (aminoglycosides, carbapenems, fluoroquinolones, penicillins + β-lactamase inhibitors, cephalosporins, folate pathway inhibitors, polymyxins and tetracyclines) were classified as MDR based on the definition of Magiorakos et al. [33] for *Acinetobacter* spp. For sulfisoxazole, cefoperazone, lomefloxacin, ticarcillin, chloramphenicol, carbenicillin and aztreonam, no breakpoints were available.

### 2.7. Statistical Analysis

In order to provide confidence intervals (CIs) for prevalence results, the Clopper–Pearson method (Binomial test) was employed using the GenBinomApps R-package (https://CRAN.R-project.org/package=GenBinomApps (accessed on 16 March 2026)). Statistical comparisons between slaughterhouse and meat processing plant prevalences, as well as between different types of industries (beef, chicken and pork) and types of samples, were performed using Fisher’s exact test by RStudio (v.2023.06.0).

## 3. Results

### 3.1. Occurrence of Acinetobacter spp.

#### 3.1.1. Occurrence in Slaughterhouses

**Figure 1:**
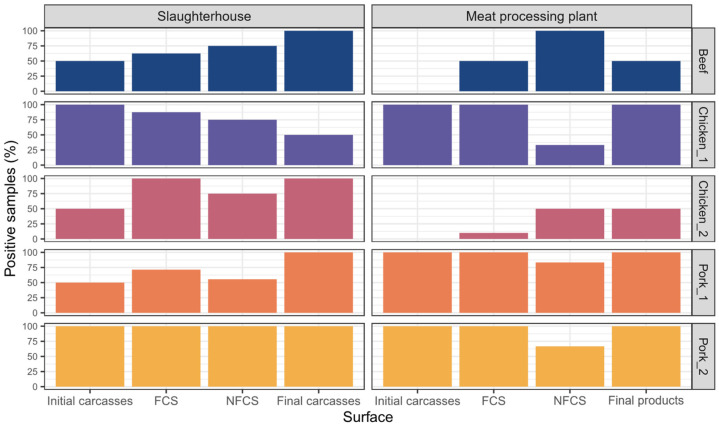
Prevalence of mesophilic and/or psychrotolerant *Acinetobacter* spp. on the different surfaces sampled in slaughterhouses and processing plants. In slaughterhouses, ‘initial carcasses’ are those taken at the end of the dirty zone, and ‘final carcasses’ are those taken at the end of the clean zone. In processing plants, ‘initial carcasses’ are sampled at the beginning of the processing process, and ‘final products’ correspond to the end meat cuts (e.g., loin, sirloin, wings, etc.).

In total, 80 out of 100 (80%, 95% CI = 71–87%) samples collected from the five slaughterhouses were positive for the presence of mesophilic and/or psychrotolerant *Acinetobacter* spp. isolates and all were negative to carbapenem-resistant *Acinetobacter* (Table 1). The prevalence of *Acinetobacter* spp. was 65% (13/20; 95% CI = 41–85%), 70% (14/20; 95% CI = 46–88%), 80% (16/20; 95% CI = 56–94%), 85% (17/20; 95% CI = 62–97%) and 100% (20/20; 95% CI = 83–100%) in the pork_1, beef, chicken_1, chicken_2, and pork_2 slaughterhouses, respectively. No statistically significant differences (*p*-value = 0.47) in prevalence were observed among slaughterhouse types (beef: 70%, 14/20, 95% CI = 46–88%; pork: 82.5%, 33/40, 95% CI = 67–93%; and chicken: 82.5%, 33/40, 95% CI = 67–93%). Prevalence across the different types of analysed surfaces was generally similar (Figure 1), without statistically significant differences (FCSs: 84.6%, 33/39, 95% CI = 69–94%; NFCSs: 75.6%, 31/41, 95% CI = 60–88%; raw materials: 80%, 16/20, 95% CI = 56–94%) (*p* = 0.65). Pork_2 exhibited a 100% prevalence across all sample types, including FCSs, NFCSs and carcasses (Figure 1). Statistically significant differences were not observed either (*p* = 0.59) between the prevalence of carcasses at the end of the dirty zone (70%, 7/10, 95% CI = 35–93%) and at the end of the clean zone (90%, 9/10, 95% CI = 55–100%) (Figure 1).

**Figure 2:**
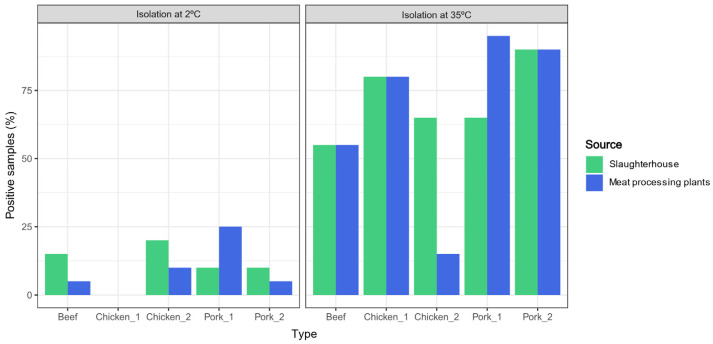
Percentage of positive samples obtained from the slaughterhouses and processing plants. Green bars represent samples collected in slaughterhouses, while blue bars represent samples collected in processing plants.

After pre-enrichment and cultivation at 35 °C, *Acinetobacter* spp. were isolated from 71 out of 100 samples (71%, 95% CI = 61–80%), with prevalence across slaughterhouses ranging from 55% (11/20; 95% CI = 32–77%; beef) to 90% (18/20; 95% CI = 68–99%; pork_2) (Figure 2). Psychrotolerant *Acinetobacter* spp., recovered at refrigeration temperatures, were detected in 11 out of 100 samples (11%; 95% CI = 5.6–19%). In this case, prevalence ranged from 0% (95% CI = 0–17%) in one chicken slaughterhouse (chicken_1) to 20% (4/20; 95% CI = 5.7–44%) in the other chicken slaughterhouse (chicken_2) (Figure 2).

#### 3.1.2. Occurrence in Meat Processing Plants

The prevalence of *Acinetobacter* spp. in the processing plants was comparable to that observed in slaughterhouses, without statistically significant differences (*p* = 0.14). In this case, 70 out of 100 (70%; 95% CI = 60–79%) samples tested positive for mesophilic and/or psychrotolerant *Acinetobacter* spp., and no carbapenem-resistant *Acinetobacter* was detected (Table 2). Detection rates were 25% (5/20; 95% CI = 8.6–49%), 60% (12/20; 95% CI =36–81%), 80% (16/20; 95% CI = 56–94%), 90% (18/20; 95% CI = 68–99%) and 95% (19/20; 95% CI = 75–100%) in samples from chicken_2, beef, chicken_1, pork_2, and pork_1 processing plants, respectively. In contrast to slaughterhouses, prevalence was significantly higher in pork (92.5%, 37/40; 95% CI = 80–98%) than in chicken (52.5%, 21/40; 95% CI = 36–68%) (*p* = 0.0001) and beef (60%, 12/20; 95% CI = 36–81%) (*p* = 0.004) processing plants. Among the FCSs (72%, 36/50; 95% CI = 58–84%), NFCSs (66.7%, 20/30; 95% CI = 47–83%) and raw materials (70%, 14/20; 95% CI = 46–88%) no significant differences were observed (*p* = 0.92), as well as between carcasses at the start of the processing line (60%, 6/10; 95% CI = 26–88%) and final meat cuts (80%, 8/10; 95% CI = 44–97%) (*p* = 0.63).

After pre-enrichment and cultivation at 35 °C, *Acinetobacter* spp. were recovered from 67% (67/100) of the samples, with prevalence ranging from 15% (3/20; 95% CI = 3–38%; chicken_2) to 95% (19/20; 95% CI = 75–100%; pork_1) (Figure 2). In general, mesophilic *Acinetobacter* prevalence in processing plants was similar to that observed in the corresponding slaughterhouses. However, two exceptions were identified: a statistically lower prevalence in the chicken_2 processing plant than in the corresponding slaughterhouse (*p* = 0.0003) and a significantly high prevalence in the processing plant relative to the slaughter line in pork_1 (*p* = 0.04) (Figure 2). Psychrotolerant *Acinetobacter* spp. were detected in 9 out of the 100 (9%; 95% CI = 4–16%) of processing plant samples. No isolates were recovered from the chicken_1 processing plant, while the highest prevalence was found in pork_1 (25%; 5/20; 95% CI = 8.6–49%) (Figure 2).

### 3.2. Diversity of Acinetobacter spp. in Meat Processing Environments

A total of 244 isolates, identified by MALDI-TOF MS as *Acinetobacter* spp. (log score value > 1.70), in accordance with Bruker’s specifications and the available literature [34,35,36], were obtained from the culture medium incubated at 35 °C. Of these isolates, four were not identified at the species level (log scores between 1.83 and 1.99). The rest of the isolates were identified with log scores ≥ 2.00 as members of the species *A. baumannii*, *A. bereziniae*, *A. calcoaceticus*, *A. courvalinii*, *A. defluvii*, *A. gandensis*, *A. gerneri*, *A. guillouiae*, *A. haemolyticus*, *A. johnsonii*, *A. junii*, *A. lactucae*, *A. nosocomialis*, *A. pittii*, *A. schindleri*, *A. seifertii*, *A. towneri*, *A. ursingii*, and *A. variabilis.* On the other hand, a total of 21 psychrotolerant *Acinetobacter* isolates were collected. These isolates were previously identified by MALDI-TOF MS as members of the genus *Acinetobacter*, and the species was subsequently confirmed by *16S rRNA* gene Sanger sequencing analysis. The difficult identification by MALDI-TOF MS of some of the psychrotolerant isolates can be attributed to the fact that they belonged to species that were not included in the MALDI-TOF database employed. The psychrotolerant isolates were identified as *A. albensis*, *A. bohemicus*, *A. harbinensis*, *A. johnsonii*, *A. lanii*, *A. pullicarnis*, *A. silvestris* and *A. terrestris.*

A total of 22 different *Acinetobacter* species were identified in samples from slaughterhouses, whereas 16 species were detected in the processing plants (Table 1 and Table 2). In both facility types, *A. baumannii* was the most prevalent species, being present in 45% (45/100) and 49% (49/100) of the samples collected from slaughterhouses and processing plants, respectively. This species was recovered from all industry types analysed. The remaining *Acinetobacter* species were found at a lower prevalence (<5% of samples) and included *A. seifertii* (4.5%; 9/200), *A. ursingii* (4.5%; 9/200), and *A. pittii* (4%; 8/200), among others. It is worth highlighting that certain species were exclusively associated with particular environments. *A. gandensis*, *A. defluvii*, *A. junii*, *A. schindleri*, *A. haemolyticus*, *A. variabilis*, *A. towneri*, *A. gerneri*, *A. lactucae*, *A. bohemicus*, and *A. terrestris* were only observed in slaughterhouse samples, whereas *A. courvalinii*, *A. guillouiae*, *A. calcoaceticus*, *A. lanii*, and *A. pullicarnis* were exclusively detected in processing plants. Additionally, *A. albensis*, *A. harbinensis*, *A. bohemicus*, *A. silvestris*, *A. lanii*, *A. terrestris*, and *A. pullicarnis* were exclusively recovered at refrigeration temperatures, indicating their psychrotolerant nature.

### 3.3. Genome Characterisation

**Figure 3:**
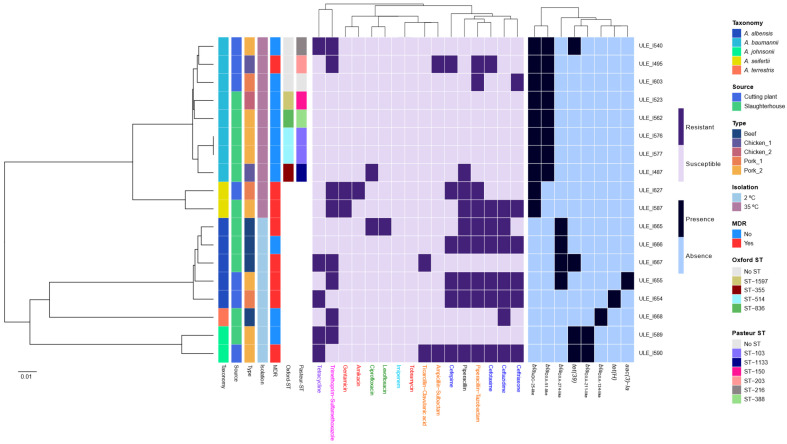
Dendrogram based on the Average Nucleotide Identity (ANI) of the 18 sequenced *Acinetobacter* spp. isolates. From left to right, the first column indicates the taxonomy, followed by the source, facility type, isolation temperature, multidrug-resistant (MDR) status, and sequence types (Oxford and Pasteur schemes, only for *A. baumannii* isolates). The heatmaps display the antimicrobial resistance phenotype (resistant, dark purple; susceptible, light purple) and the presence (dark blue) or absence (light blue) of ARGs. All *tet*(*39*) genes were located on plasmidic contigs. Antimicrobial categories used to define MDR, according to Magiorakos et al. [33]: Aminoglycosides—red colour; carbapenems—light blue colour; fluoroquinolones—green colour; penicillins + β-lactamase inhibitors—orange colour; cephalosporins—blue colour; folate pathway inhibitors—pink colour; tetracyclines—purple colour.

The species identification of the 18 sequenced isolates (eight *A. baumannii*, two *A. seifertii*, five *A. albensis*, two *A. johnsonii*, and one *A. terrestris*), initially obtained by MALDI-TOF MS or *16S rRNA* gene Sanger sequencing, was confirmed by GTDB-Tk. The analysis of the genome sequences revealed the presence of various ARGs characteristic of *Acinetobacter* spp., including OXA-type beta-lactamase genes, such as *bla*_OXA-51-like_, *bla*_OXA-211-like_, *bla*_OXA-274-like_ and *bla*_OXA-134-like_ allelic variants, and the ADC-type chromosomal cephalosporinase gene *bla*_ADC-25-like_ (Figure 3). In particular, all *A. baumannii* isolates harboured *bla*_OXA-51-like_ and *bla*_ADC-25-like_ genes, with the latter also being detected in all *A. seifertii* genomes. *bla*_OXA-211-like_ genes were identified in all *A. johnsonii* isolates, whereas a *bla*_OXA-134-like_ gene was observed in the single *A. terrestris* genome. In addition, *bla*_OXA-274-like_ genes were present in four out of five (80%) *A. albensis* genomes. Acquired ARGs linked with resistance to aminoglycosides and tetracyclines were identified in six isolates (6/18; 33.3%) (Figure 3). Tetracycline resistance genes included *tet*(*39*), identified in two *A. johnsonii*, one *A. albensis* and one *A. baumannii* isolate; and *tet*(*H*), detected in one *A. albensis* genome. The aminoglycoside resistance gene *aac*(*3*)*-Ia* was identified in one *A. albensis* isolate. Of these genes, all *tet*(*39*) sequences were located in plasmidic contigs. Additionally, a *gyrA* (L81S) mutation was identified in one of the fluoroquinolone-resistant isolates from *A. albensis* (ULE_I665).

### 3.4. MLST of A. baumannii Isolates

MLST analysis of *A. baumannii* isolates revealed four (ST-355, ST-1597, ST-836, and ST-514) and six (ST-1133, ST-203, ST-150, ST-216, ST-388, and ST-103) different known sequence types (STs) using the Oxford and Pasteur schemes, respectively (Figure 3). Only one *A. baumannii* isolate (ULE_I603) could not be assigned to any known ST, whereas two isolates (ULE_I576 and ULE_I577), collected from different samples within the same slaughterhouse (pork_2), were identified as belonging to the same STs: ST-514 (Oxf) and ST-103 (Pa) (Figure 3).

### 3.5. Phylogenetic Relationship with Strains from Fresh Meat

**Figure 4:**
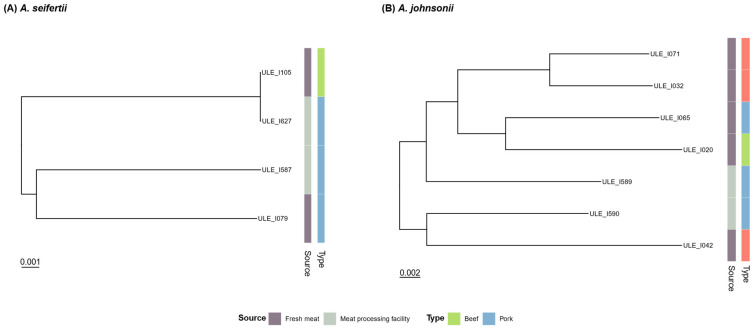
Core-genome phylogenetic trees of *A. seifertii* (**A**) and *A. johnsonii* (**B**) isolates collected from fresh meat in a previous study [13] and from meat processing facilities (slaughterhouses and processing plants) in the present work. From left to right, the first column indicates the source of isolation, and the second column specifies the type of meat or meat processing facility.

**Figure 5:**
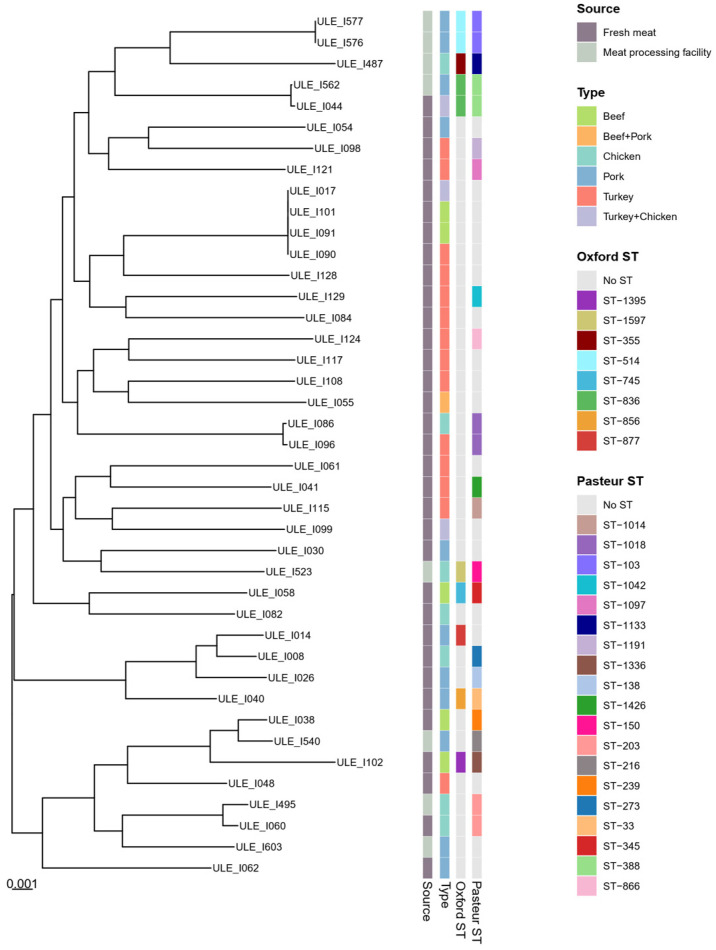
Core-genome phylogenetic tree of *A. baumannii* isolates collected from fresh meat in a previous study [13] and from meat processing facilities (slaughterhouses and processing plants). From left to right, the first column indicates the source of isolation, followed by the type of meat or meat processing facility where the isolates were obtained and their sequence types (Oxford and Pasteur schemes).

The construction of core-genome phylogenetic trees with *A. baumannii*, *A. seifertii*, and *A. johnsonii* genomes (including genomes from fresh meat and meat preparations, from Puente et al. [13]) demonstrated that certain isolates from meat processing environments and fresh meat are closely related (Figure 4 and Figure 5). Indeed, two *A. seifertii* isolates, one isolated from a pork processing facility (ULE_I627) and the other from beef meat (ULE_I105), appear to be clonally related (Figure 4), and this same observation was made for two *A. baumannii* isolates from other pig slaughterhouse (ULE_I562) and a mixed turkey-chicken meat product (ULE_I044) (Figure 5). These two *A. baumannii* isolates also belonged to the same STs (ST-836Ox and ST-388Pa), as can be observed in Figure 5. Furthermore, one *A. baumannii* collected in a chicken processing plant (ULE_I495) and another from chicken meat (ULE_I060) were also assigned to a common Pasteur ST (ST-203) (Figure 5).

### 3.6. Antimicrobial Susceptibility Profiles

The antimicrobial susceptibility testing showed that, according to EUCAST and CLSI clinical breakpoints, 14 (six mesophilic and eight psychrotolerant) out of 18 (77.8%) isolates analysed were resistant to at least one antibiotic (Figure 3 and Table S1). In particular, these isolates exhibited resistance to piperacillin-tazobactam (50%; 9/18), piperacillin (44.4%; 8/18), trimethoprim-sulfamethoxazole (44.4%; 8/18), ceftazidime (38.9%; 7/18), cefotaxime (38.9%; 7/18), ceftriaxone (33.3%, 6/18), cefepime (33.3%; 6/18), tetracycline (27.8%; 5/18), ticarcillin-clavulanic acid (11.1% 2/18), ampicillin-sulbactam (11.1%; 2/18), ciprofloxacin (11.1%; 2/18), gentamicin (11.1%; 2/18), levofloxacin (5.6%; 1/18), and amikacin (5.6%; 1/18) (Figure 3). All isolates were susceptible to imipenem and tobramycin. However, for sulfisoxazole, cefoperazone, lomefloxacin, ticarcillin, chloramphenicol, carbenicillin and aztreonam, resistance status could not be determined, since the clinical breakpoints for these antibiotics are not available. The MIC values for these antimicrobials are shown in Table S1. In general, psychrotolerant *Acinetobacter* spp. demonstrated higher levels of resistance compared to mesophilic isolates (Figure 3). Furthermore, 44.4% (8/18) of the *Acinetobacter* spp. isolates (3/10 mesophilic and 5/8 psychrotolerant) were classified as MDR, as they showed resistance to at least one antibiotic in three or more antimicrobial categories (Figure 3). Conversely, 22.2% (4/18) of the isolates were susceptible to all antibiotics tested.

## 4. Discussion

Members of the genus *Acinetobacter* are widely recognised as common inhabitants of food processing environments, where they can persist on equipment surfaces and in residues due to their remarkable adaptability. Studies using whole-metagenome sequencing have repeatedly identified *Acinetobacter* among the predominant genera in meat processing facilities [7,10,37,38,39,40]. In this context, the frequent occurrence of these bacteria within slaughterhouses (80%, 95% CI = 71–87%) and processing plants (70%, 95% CI = 60–79%) observed in the present study highlights their potential role as reservoirs in the meat production chain. In contrast, Chung et al. [41] observed a much lower prevalence of *A. baumannii* in carcasses and environmental samples from pig and cattle slaughterhouses in South Korea, ranging from 0% to 20.8%. The lower prevalence values reported by Chung et al. [41], compared to those observed in the present study, may be explained by the fact that their investigation focused solely on a single species, *A. baumannii*, as well as the different samples being analysed (carcasses, knives, processing boards, floors and toilets), among other possible reasons. The presence of *Acinetobacter* along slaughter lines and corresponding processing plants could be due to various factors. Live animals entering slaughterhouses could act as carriers of microorganisms present on their skin from soil, water and faecal sources [42]. *Acinetobacter* spp. are ubiquitous and widely distributed in environmental reservoirs such as soil, water, sewage, as well as in animals and humans [43]. In addition, slaughterhouse personnel may also act as a reservoir, as *Acinetobacter* can colonise human skin [43,44]. This potential route of transmission was supported by Chung et al. [41], who identified an identical *A. baumannii* ST (ST-3500) in both carcasses and workers in a cattle slaughterhouse in South Korea. Moreover, some *Acinetobacter* spp. isolates from meat processing environments in this study were phylogenetically related to others previously obtained from fresh meat (e.g., pork processing facility—beef meat, pig slaughterhouse—mixed turkey-chicken meat product). These observations could indicate a possible circulation of *Acinetobacter* spp. among different animal production chains. Specifically, MLST analysis revealed two distinct STs, ST-203 (Pa) and ST-836 (Oxf)/ST-388 (Pa), in both meat processing environments and fresh meat samples. ST-836 (Oxf)/ST-388 (Pa) was also previously found in a poultry slaughterhouse in Germany, particularly in a sewage water sample [45]. In addition, the ST-1597 (Pa) detected in our study in one chicken slaughterhouse was previously detected in a pig slaughterhouse in South Korea [41]. The ST-355 (Oxf) isolated from the other chicken slaughterhouse analysed in our study was also detected in faecal samples from sick ducks, pigs, chickens and geese in China [46], as well as in bronchoalveolar fluid from a Chinese hospital patient [47]. Although some of the STs described in this study have been detected in clinical samples worldwide (e.g., ST-216 (Pa) in Canadian and Iranian hospitals) [48,49,50], they do not belong to the “international clones of high risk” (ICs), a range of lineages associated with antimicrobial resistance and global dissemination [51]. Nevertheless, despite this evidence of genomic similarity between meat isolates, isolates from meat processing environments, and clinical isolates, it is not possible to ascertain the direction of transmission.

The prevalence of *Acinetobacter* spp. reported herein was high regardless of the surface being analysed. Tsitsos et al. [52] also detected *Acinetobacter* spp. in FCSs and NFCSs of two poultry slaughterhouses from Greece. The frequent detection on processing surfaces is likely related to the high tolerance of *Acinetobacter* to disinfectants and its ability to form biofilms [6,53,54,55,56]. Carcasses sampled at the end of the slaughter line and the final meat cuts showed a slightly higher *Acinetobacter* prevalence than carcasses at the initial stages of the slaughtering or processing processes, although this difference was not statistically significant. These findings are consistent with Fernández-Trapote et al. [10], who found an increase in the relative abundance of *Acinetobacter*, in particular *A. johnsonii*, in carcasses from intermediate and final slaughter stages, using a metagenomic approach. The analysis of different industry sectors, namely beef, pork and chicken, allowed the identification of slaughterhouses and meat processing plants as reservoirs of *Acinetobacter* spp., regardless of the type of processed meat. The prevalence observed in pork processing plants was significantly higher than that detected in beef and poultry facilities; however, this result should be interpreted with caution. The number of samples collected was not the same for all the different types of facilities, and the study of only five industries could have influenced the observed differences. Moreover, the lower prevalence of *Acinetobacter* spp. in one of the pig slaughterhouses studied than in the corresponding processing plant might be linked to the cleaning effectiveness, resulting in greater bacterial reduction [8,57]. This explanation may also account for the absence of psychrotolerant *Acinetobacter* spp. isolates in one of the chicken facilities, and for the low prevalence of mesophilic isolates in the processing plant of the other chicken facility. Although no data could be obtained on the hygiene or sanitation practices followed at the analysed facilities, it is reasonable to assume that the presence of *Acinetobacter* spp. in processing environments can be influenced by hygiene measures, and that dedicated cleaning and disinfection of equipment, adequate handling practices, and pro-active environmental monitoring programmes, among other activities, may contribute to reduce the dissemination of this bacterium throughout the meat processing chain.

A wide diversity of *Acinetobacter* species was observed, particularly in samples from slaughterhouses. In total, 22 species were identified in slaughterhouses by MALDI-TOF MS and/or *16S rRNA* analysis, while in the meat processing plants, 16 different species were detected. Among all the identified species, some have been linked in the past to nosocomial infections, including *A. baumannii* (present in around 50% of the samples), *A. nosocomialis*, *A. pitti*, *A. junii*, *A. bereziniae*, *A. johnsonii*, and *A. ursingii* [14,17,18]. These findings agree with those of the study conducted by Tsitsos et al. [52], who observed that of the 38 *Acinetobacter* spp. isolates collected from slaughterhouse environments, 37 were identified as *A. baumannii* and only one as *A. pittii*. Furthermore, we detected both psychrotolerant and mesophilic *Acinetobacter* spp. isolates. Considering the capacity of some *Acinetobacter* species to grow at low temperatures typical of meat storage rooms and processing plants [6], a higher prevalence of psychrotolerant *Acinetobacter* could have been expected than that found in the current study. Indeed, a previous study characterising the microbiome of Spanish meat processing facilities by whole-metagenome sequencing reported one of the psychrotolerant *Acinetobacter* species here isolated, *A. harbinensis*, among the most abundant bacterial species within these environments [40]. The lower amount of psychrotolerant strains obtained, compared to mesophilic ones, could be attributed to the fact that psychrotolerant species were isolated through a direct spread plating method, without a pre-enrichment step, while mesophilic strains underwent a non-selective pre-enrichment step prior to isolation. In addition, the low incubation temperature employed (2 °C) may have contributed to a lower recovery of psychrotolerant *Acinetobacter* spp.

The genomic characterisation of 18 *Acinetobacter* spp. isolates revealed ARGs conferring resistance to beta-lactams, tetracyclines, and/or aminoglycosides in all the sequenced strains. These findings are in alignment with previous studies identifying beta-lactam, tetracycline and aminoglycoside resistance genes as the most abundant ARG families in meat processing environments [7,39,40]. The *tet*(*39*) was the most frequently detected acquired ARG, identified in four isolates and the only one located on plasmid contigs. Similarly, Fernández-Trapote et al. [10] identified *tet*(*39*) as the main ARG detected in carcasses and surfaces from a pork slaughterhouse and processing plant, where it was frequently located on plasmids.

Antimicrobial susceptibility testing revealed the resistance of various *Acinetobacter* strains to several antibiotics. Nevertheless, it is important to note that all *Acinetobacter* spp. isolates analysed in this study exhibited susceptibility to carbapenems, last-resort antibiotics used for treating MDR infections. These findings are consistent with observations reported by Tsitsos et al. [58], Chung et al. [41], and Hamouda et al. [59], who identified that *Acinetobacter* spp. isolated from carcasses and/or slaughterhouses demonstrated sensitivity to carbapenem antibiotics, specifically imipenem and meropenem. Moreover, this susceptibility profile aligns with the absence of isolates obtained on the selective medium used for the detection of carbapenem-resistant strains. In general, the beta-lactam resistance observed among the isolates tested could be attributed to the presence of *bla*_OXA-type_ and *bla*_ADC-25-like_ genes. Among the *bla*_OXA_ group we identified *bla*_OXA-51-like_ genes, the biggest group of OXA-type beta-lactamases, which are naturally found in *A. baumannii* [60]; *bla*_OXA-211-like_ genes, natural in *A. johnsonii; bla*_OXA-274-like_, previously detected in *A. guillouiae*, *A. kanungonis*, *A. tandoii*, and *A. stercoris* [61], whereas in the present study it was detected in four out of five *A. albensis* isolates; and *bla*_OXA-134-like_ genes, previously found in *A. lwoffii*, *A. baumannii*, *A. schindleri*, and *A. pseudolwoffii* [61], while in the current study was detected in *A. terrestris.* Nevertheless, not all isolates harbouring *bla*_OXA-type_ and/or *bla*_ADC-25-like_ genes showed beta-lactam resistance, as demonstrated by various pansusceptible *A. baumannii* isolates. This observation may be attributed to the fact that these intrinsic genes confer resistance primarily when overexpressed, for instance, when an insertion sequence such as *ISAba1* is located upstream of the gene [60,62]. Conversely, one *A. albensis* isolate was found to be resistant to various beta-lactams despite lacking known ARGs. Similarly, Tsitsos et al. [58] found that 57.6% of *Acinetobacter* isolates from poultry carcasses in two slaughterhouses were resistant to ceftazidime, cefotaxime, piperacillin and piperacillin-tazobactam, but only 10.2% carried a known beta-lactamase gene. Another study from Tsitsos et al. [52] also reported that only 10 out of 41 ceftazidime-resistant *Acinetobacter* strains isolated from slaughterhouse environments carried beta-lactamase genes. On the other hand, the tetracycline resistance observed for five isolates could be associated with the presence of *tet*(*39*) or *tet*(*H*) genes [63]. Moreover, the *gyrA* (S81L) mutation, associated with fluoroquinolone resistance, in one *A. albensis* strain could be responsible for the observed resistance to ciprofloxacin and levofloxacin [64,65]. Considering that *Acinetobacter* spp. are intrinsically resistant to trimethoprim according to EUCAST expert rules, the presence of some isolates with phenotypic resistance to trimethoprim-sulfamethoxazole was not unexpected [66]. Finally, resistance to aminoglycosides (gentamicin and/or amikacin) in some isolates might be linked to *16S rRNA* or ribosomal protein mutations [67,68], as no associated ARGs were detected. However, the *aac*(*3*)*-Ia* gene present in one *A. albensis* strain did not confer aminoglycoside resistance. Overall, 44.4% (8/18) of the isolates were classified as MDR based on the definition of Magiorakos et al. [33]. Two Greek studies documented an even higher percentage, with 88.1% of *Acinetobacter* spp. from poultry carcasses and 87.8% from poultry slaughterhouse environments exhibiting MDR phenotypes [42,58]. The MDR trait is particularly prevalent in this genus, especially in *A. baumannii* [69].

It is important to note that the current study is not a longitudinal survey, and therefore, the results reflect the point prevalence at the time of sampling. The assessment of the environmental persistence of *Acinetobacter* spp. would require repeated sampling over time, including different seasons and a larger number of industries. Although three different animal production chains were included in our study, only five industries were sampled, which may limit the impact of the results obtained. Moreover, no information could be obtained regarding facility-specific practices or cleaning and disinfection protocols, factors that could influence the occurrence of *Acinetobacter* spp. Overall, these limitations should be considered when interpreting the findings, particularly in relation to differences in prevalence among facilities and to the potential persistence of *Acinetobacter* spp. in the studied environments.

## 5. Conclusions

To the best of our knowledge, this is the first study reporting the prevalence, diversity of species, and AMR profile of *Acinetobacter* spp. from slaughterhouses and meat processing plants in Spain. Our findings suggest that meat processing environments may act as reservoirs for a diverse array of mesophilic and psychrotolerant *Acinetobacter* species, especially *A. baumannii*. The phylogenetic analyses indicated a possible circulation of *Acinetobacter* isolates between fresh meat and meat processing environments. Moreover, the presence of isolates resistant to beta-lactams, tetracyclines, fluoroquinolones, aminoglycosides, and folate pathway antagonists, several of which showed MDR phenotypes, as well as the detection of ARGs conferring resistance to beta-lactams, tetracyclines, and aminoglycosides, suggests that these environments could contribute to the dissemination of AMR. These findings underscore the need to implement appropriate hygiene and control measures to reduce the contamination and spread of resistant strains throughout the meat processing chain, and evidence the need for further research to better understand the potential role of meat processing environments in AMR transmission.

## Figures and Tables

**Table 1:** Characteristics of samples collected from slaughterhouses, including the number of samples analysed, number of *Acinetobacter*-positive samples at isolation temperatures of 35 °C and 2 °C, and *Acinetobacter* species identified by MALDI-TOF MS (log score ≥ 2) or *16S rRNA* gene sequencing (indicated in bold). Isolates with MALDI-TOF log scores < 2 are indicated as *Acinetobacter* spp. Grey shading indicates chicken_1 and pork_1 samples; unshaded columns (close to shaded ones) correspond to chicken_2 and pork_2 samples.

Industry Type	Surfaces		Samples		Positive Samples				*Acinetobacter* Species			
					35 °C		2 °C		35 °C		2 °C	
BEEF	FCS	Knives	5		3		0		*A. baumannii*, *A. variabilis*, *Acinetobacter* spp.		*-*	
		Hook	1		0		1		*-*		***A. albensis***	
		Trays	2		1		0		*A. haemolyticus*		*-*	
	NFCS	Drains	4		3		0		*Acinetobacter* spp., *A. baumannii*, *A. gandensis*, *A. variabilis*		*-*	
		Floors	2		2		0		*A. schindleri*, *A. gandensis*		*-*	
		Walls	2		0		1		*-*		***A. terrestris***	
	Carcasses	Start	2		0		1		*-*		***A. albensis***	
		Final	2		2		0		*A. baumannii*		*-*	
CHICKEN	FCS	Scalding water	1	1	1	1	0	0	*A. baumannii*	*A. baumannii*	*-*	*-*
		Hook	1	1	1	1	0	0	*A. baumannii*	*A. baumannii*	*-*	*-*
		Knives	4	4	3	3	0	1	*A. baumannii*, *A. johnsonii*	*A. baumannii*, *A. junii*	*-*	***A. harbinensis***
		Trays	2	2	2	0	0	2	*A. baumannii*, *A. bereziniae*, *A. pittii*	*-*	*-*	***A. silvestris*** **,** ***A. harbinensis***
	NFCS	Drains	4	4	3	3	0	0	*A. baumannii*, *A. gerneri*, *A. bereziniae*, *A. lactucae*	*A. baumannii*	*-*	*-*
		Floors	2	2	2	2	0	0	*A. baumannii*, *A. defluvii*	*A. baumannii*, *A. pittii*	*-*	*-*
		Walls	2	2	1	0	0	1	*A. baumannii*	*-*	*-*	***A. harbinensis***
	Carcasses	Start	2	2	2	1	0	0	*A. baumannii*	*A. nosocomialis*	*-*	*-*
		Final	2	2	1	2	0	0	*A. baumannii*	*A. baumannii*, *A. nosocomialis*	*-*	*-*
PORK	FCS	Scalding water	1	1	1	1	0	0	*A. baumannii*	*A. baumannii*	*-*	*-*
		De-hairing	1	1	1	1	1	0	*A. baumannii*	*A. pittii*	***A. bohemicus***	*-*
		Knives	3	4	3	4	0	0	*A. gandensis*, *A. defluvii*, *A. junii*, *A. ursingii*	*A. baumannii*, *A. seifertii*, *A. nosocomialis*	*-*	*-*
		Trays	2	2	0	2	0	0	*-*	*A. baumannii*, *A. pittii*	*-*	*-*
	NFCS	Drains	5	4	3	4	0	0	*A. johnsonii*, *A. pittii*, *A. towneri*, *Acinetobacter* spp.	*A. baumannii*, *A. nosocomialis*	*-*	*-*
		Floors	2	2	1	2	1	0	*A. baumannii*	*A. baumannii*, *A. pittii*	***A. bohemicus***	*-*
		Walls	2	2	1	2	0	0	*A. schindleri*	*A. baumannii*, *A. haemolyticus*	*-*	*-*
	Carcasses	Start	2	2	1	2	0	0	*A. johnsonii*	*A. baumannii*	*-*	*-*
		Final	2	2	2	0	0	2	*A. baumannii*, *A. ursingii*	*-*	*-*	*A. johnsonii*

**Table 2:** Characteristics of samples collected from processing plants, including the number of samples analysed, number of *Acinetobacter*-positive samples at isolation temperatures of 35 °C and 2 °C, and *Acinetobacter* species identified by MALDI-TOF MS (log score ≥ 2) or *16S rRNA* gene sequencing (indicated in bold). Isolates with MALDI-TOF log scores < 2 are indicated as *Acinetobacter* spp. Grey shading indicates chicken_1 and pork_1 samples; unshaded columns (close to shaded ones) correspond to chicken_2 and pork_2 samples.

Industry Type	Surfaces		Samples		Positive Samples				*Acinetobacter* Species			
					35 °C		2 °C		35 °C		2 °C	
BEEF	FCS	Knives	3		1		0		*A. courvalinii*		*-*	
		Conveyor belts	3		3		0		*A. courvalinii*		*-*	
		Trays	2		0		0		*-*		*-*	
		Cutting boards	2		1		0		*A. courvalinii*		*-*	
	NFCS	Drains	2		2		0		*A. baumannii*		*-*	
		Floors	2		2		0		*A. courvalinii*, *Acinetobacter* spp.		*-*	
		Walls	2		1		1		*Acinetobacter* spp.		***A. lanii***	
	Carcasses/meat cuts	Start	2		0		0		-		-	
		Final product	2		1		0		*A. courvalinii*		-	
CHICKEN	FCS	Knives	3	3	3	0	0	0	*A. baumannii*, *A. johnsonii*	-	*-*	-
		Conveyor belts	3	3	3	0	0	0	*A. baumannii*, *A. pittii*	-	*-*	-
		Trays	2	2	2	0	0	1	*A. baumannii*	-	*-*	***A. harbinensis***
		Cutting boards	2	2	2	0	0	0	*A. baumannii*	-	*-*	-
	NFCS	Drains	2	2	0	1	0	0	*-*	*A. calcoaceticus*	*-*	*-*
		Floors	2	2	1	1	0	0	*A. baumannii*	*A. baumannii*	*-*	*-*
		Walls	2	2	1	1	0	0	*A. baumannii*	*Acinetobacter* spp.	*-*	*-*
	Carcasses/meat cuts	Start	2	2	2	0	0	0	*A. baumannii*	-	-	-
		Final product	2	2	2	0	0	1	*A. baumannii*	-	-	***A. harbinensis***
PORK	FCS	Knives	3	3	3	3	0	0	*A. baumannii*, *A. ursingii*, *A. seifertii*	*A. baumannii*, *A. ursingii*	*-*	*-*
		Conveyor belts	3	3	3	3	2	0	*A. baumannii*, *A. nosocomialis*, *A. seifertii*, *A. ursingii*, *A. bereziniae*	*A. baumannii*, *A. seifertii*, *A. ursingii*	***A. lanii*** **,** ***A. silvestris***	*-*
		Trays	2	2	2	2	1	0	*A. baumannii*	*A. baumannii*	***A. pullicarnis***	*-*
		Cutting boards	2	2	2	2	0	1	*A. baumannii*, *A. seifertii*	*A. baumannii*	*-*	*A. johnsonii*
	NFCS	Drains	2	2	2	2	0	0	*A. baumannii*	*A. baumannii*, *A. bereziniae*	*-*	*-*
		Floors	2	2	2	2	0	0	*A. baumannii*, *A. ursingii*	*A. baumannii*, *A. bereziniae*, *A. seifertii*	*-*	*-*
		Walls	2	2	1	0	0	0	*A. guillouiae*	-	*-*	-
	Carcasses/meat cuts	Start	2	2	2	2	1	0	*A. baumannii*, *A. ursingii*	*A. baumannii*	***A. albensis***	*-*
		Final product	2	2	2	2	1	0	*A. baumannii*, *A. bereziniae*, *A. seifertii*	*A. baumannii*	***A. albensis***	*-*

## Data Availability

Genome fasta files obtained in the current study are available at the National Center of Biotechnology Information (NCBI) under the BioProject accession number PRJNA1417964.

## Supplementary Materials

The following supporting information can be downloaded at: https://www.mdpi.com/article/10.3390/foods15071243/s1; Table S1: MICs (µg/mL) of the antibiotics analysed and ARGs detected in 18 *Acinetobacter* spp. isolates.

## Abbreviations

The following abbreviations are used in this manuscript:

AMR	Antimicrobial resistance
ANI	Average Nucleotide Identity
ARGs	Antimicrobial resistance genes
BHI	Brain–heart infusion
BPW	Buffered peptone water
CLSI	Clinical and Laboratory Standards Institute
ESKAPE	*Enterococcus faecium*, *Staphylococcus aureus*, *Klebsiella pneumoniae*, *Acinetobacter baumannii*, *Pseudomonas aeruginosa* and *Enterobacter* spp.
EUCAST	European Committee on Antimicrobial Susceptibility Testing
FCS	Food contact surface
MALDI-TOF MS	Matrix-Assisted Laser Desorption–Ionisation Time-of-Flight Mass Spectrometry
MDR	Multidrug-resistant
MIC	Minimum inhibitory concentration
MLST	Multi-locus sequence typing
NFCS	Non-food contact surface
NCBI	National Center for Biotechnology Information
STs	Sequence types
TFA	Tri-fluoroacetic-acid
WGS	Whole-genome sequencing
WHO	World Health Organization
