# Supplementary material for: Occurrence and Antimicrobial Resistance of Acinetobacter spp. in Processing Environments of Slaughterhouses and Meat Processing Facilities
Source: Foods. 2026 Apr 5;15(7):1243. doi: 10.3390/foods15071243 (PMC13074205; doi:10.3390/foods15071243)
Supplement: Supplementary file 1 [file foods-15-01243-s001.zip › foods-4193371-supplementary.pdf]

Table S1. MICs (µg/mL) of the antibiotics analysed and ARGs detected in 18 *Acinetobacter* spp. isolates. ARGs: antimicrobial resistance genes.

	TAZ	LEVO	FIS	PIP	P/T4	AMI	FOP	LOM	IMI	TIM2	TIC	SXT	A/S2	FOT	CHL	TOB	AXO	FEP	CAR	AZT	CIP	TET	GEN	ARGs
**ULE_I487**	16	0.25	≤256	>64	32/4	≤4	>32	≤0.5	≤1	32/2	32	≤0.5/9.5	16/8	16	>16	≤1	16	4	256	>16	2	2	≤1	*bla*$_{ADC-25-like}$, *bla*$_{OXA-51-like}$
**ULE_I495**	8	0.25	>256	64	>64/4	≤4	>32	≤0.5	≤1	32/2	>64	8/152	>16/8	>32	>16	≤1	16	>16	64	>16	≤0.25	2	≤1	*bla*$_{ADC-25-like}$, *bla*$_{OXA-51-like}$
**ULE_I523**	8	≤0.12	≤256	64	32/4	≤4	>32	≤0.5	≤1	32/2	32	≤0.5/9.5	8/4	16	>16	≤1	16	4	64	>16	≤0.25	≤1	≤1	*bla*$_{ADC-25-like}$, *bla*$_{OXA-51-like}$
**ULE_I540**	8	≤0.12	>256	16	≤8/4	≤4	32	≤0.5	≤1	≤16/2	32	>4/76	4/2	16	>16	≤1	16	≤2	128	>16	≤0.25	>8	≤1	*bla*$_{ADC-25-like}$, *bla*$_{OXA-51-like}$, *tet(39)*
**ULE_I562**	16	≤0.12	≤256	32	16/4	≤4	>32	1	≤1	64/2	32	≤0.5/9.5	4/2	16	>16	≤1	32	4	64	>16	0.5	≤1	≤1	*bla*$_{ADC-25-like}$, *bla*$_{OXA-51-like}$
**ULE_I576**	4	≤0.12	≤256	16	≤8/4	≤4	>32	≤0.5	≤1	64/2	16	≤0.5/9.5	4/2	8	>16	≤1	≤4	≤2	≤32	16	≤0.25	≤1	≤1	*bla*$_{ADC-25-like}$, *bla*$_{OXA-51-like}$
**ULE_I577**	4	≤0.12	≤256	64	64/4	≤4	>32	≤0.5	≤1	32/2	32	≤0.5/9.5	8/4	8	>16	≤1	16	4	64	>16	≤0.25	≤1	≤1	*bla*$_{ADC-25-like}$, *bla*$_{OXA-51-like}$
**ULE_I587**	>16	0.25	>256	>64	>64/4	8	>32	2	≤1	64/2	>64	>4/76	16/8	>32	>16	2	>32	8	128	>16	1	2	8	*bla*$_{ADC-25-like}$
**ULE_I603**	16	≤0.12	≤256	32	>64/4	8	>32	≤0.5	≤1	64/2	64	≤0.5/9.5	8/4	32	>16	≤1	>32	8	64	>16	≤0.25	2	≤1	*bla*$_{ADC-25-like}$, *bla*$_{OXA-51-like}$
**ULE_I627**	8	0.25	>256	>64	>64/4	16	>32	2	≤1	64/2	32	1/19	8/4	32	>16	4	32	>16	64	>16	0.5	4	>8	*bla*$_{ADC-25-like}$
**ULE_I589**	16	≤0.12	>256	64	≤8/4	≤4	>32	≤0.5	≤1	≤16/2	16	1/19	≤2/1	8	16	≤1	≤4	≤2	64	>16	0.5	>8	≤1	*bla*$_{OXA-211-like}$, *tet(39)*
**ULE_I590**	>16	1	>256	>64	>64/4	8	>32	4	≤1	>128/2	>64	≤0.5/9.5	>16/8	>32	>16	≤1	>32	>16	>256	>16	1	>8	≤1	*bla*$_{OXA-211-like}$, *tet(39)*
**ULE_I654**	>16	≤0.12	≤256	>64	>64/4	≤4	>32	≤0.5	≤1	≤16/2	>64	≤0.5/9.5	16/8	>32	4	≤1	>32	>16	>256	>16	0.5	>8	≤1	*tet(H)*
**ULE_I655**	>16	≤0.12	>256	>64	>64/4	≤4	>32	≤0.5	2	≤16/2	>64	>4/76	16/8	>32	4	≤1	>32	>16	>256	>16	1	2	≤1	*bla*$_{OXA-274-like}$, *aac(3)-Ia*
**ULE_I665**	>16	8	>256	>64	>64/4	≤4	>32	8	≤1	32/2	>64	≤0.5/9.5	8/4	>32	4	≤1	32	8	256	>16	2	≤1	≤1	*bla*$_{OXA-274-like}$
**ULE_I666**	>16	≤0.12	≤256	>64	>64/4	≤4	>32	≤0.5	≤1	≤16/2	>64	≤0.5/9.5	≤2/1	>32	4	≤1	>32	>16	256	>16	≤0.25	≤1	≤1	*bla*$_{OXA-274-like}$
**ULE_I667**	8	0.5	>256	16	16/4	≤4	>32	≤0.5	≤1	>128/2	≤8	4/76	8/4	8	8	≤1	≤4	≤2	≤32	16	≤0.25	>8	≤1	*bla*$_{OXA-274-like}$, *tet(39)*
**ULE_I668**	>16	≤0.12	>256	16	≤8/4	≤4	>32	≤0.5	≤1	16/2	16	2/38	≤2/1	≤4	4	≤1	≤4	4	≤32	>16	≤0.25	≤1	≤1	*bla*$_{OXA-134-like}$

TAZ, ceftazidime; LEVO, levofloxacin; FIS, sulfisoxazole; P/T4, piperacillin/tazobactam constant 4; AMI, amikacin; FOP, cefoperazone; LOM, lomefloxacin; IMI, imipenem; TIM2, ticarcillin/clavulanic acid constant; TIC, ticarcillin; SXT, trimethoprim/sulfamethoxazole; A/S2, ampicillin/sulbactam 2:1 ratio; FOT, cefotaxime; CHL, chloramphenicol; TOB, tobramycin; AXO, ceftriaxone; FEP, cefepime; CAR, carbenicillin; AZT, aztreonam; CIP, ciprofloxacin; TET, tetracycline; GEN, gentamicin.

EUCAST clinical breakpoints for *Acinetobacter* spp. (µg/mL): LEVO, >1; AMI, >8; IMI, >4; SXT, >0.5; TOB, >4; CIP, >1; GEN, >4.

CLSI clinical breakpoints for *Acinetobacter* spp. (µg/mL): TAZ, ≥32; PIP, ≥128; P/T4, ≥128/4; TIM2, ≥128/2; A/S2, ≥32/16; FOT, ≥64; AXO, ≥64; FEP, ≥32; TET, ≥16

Clinical breakpoints no available: FIS, FOP, LOM, TIC, CHL, CAR, AZT

Antimicrobial categories used to define MDR according Magiorakos et al. [33]: Aminoglycosides – red colour; carbapenems – light blue colour; fluoroquinolones – green colour; penicillins + β-lactamase inhibitors – orange colour; cephalosporins – blue colour; folate pathway inhibitors – pink colour; tetracyclines – purple colour
